# ATP, a double-edged sword in cancer

**DOI:** 10.18632/oncoscience.230

**Published:** 2015-08-30

**Authors:** Jean X. Jiang, Manuel A. Riquelme, Jade Z. Zhou

**Affiliations:** Department of Biochemistry, University of Texas Health Science Center, San Antonio, TX, USA

**Keywords:** ATP, adenosine, breast cancer bone metastasis, purinergic receptor signaling

The anti-neoplastic activity of ATP was first demonstrated in the early 80's, where exogenous ATP inhibited the growth of pancreatic and colon cancer cells [1]. Since then, other studies have also shown the inhibitory effect of ATP on various cancers including prostate, breast, colon, liver, ovarian, colorectal, esophageal, melanoma and leukemia. Intravenous ATP increases the survival rate in clinical trials in patients with pre-terminal cancer [2]. Although multiple studies show anticancer action of ATP through its binding to P2 purinergic receptors [3], there are also reports of ATP and P2 purinergic receptor mediating pro-tumorigenic effects in prostate and breast cancer cells [4]. This discrepancy had remained unresolved until our recent paper [5] demonstrating the biphasic action of ATP on breast cancer cells. In this paper, we show that ATP released from osteocytes, the most abundant cell type in bone tissue, exerts inhibitory effects on breast cancer cells. Additionally, the use of an agonist for P2X_7_, a purinergic receptor for ATP, inhibited cancer cell growth and migration. Intriguingly, ATP at higher concentrations promoted breast cancer cell growth and migration, but this adverse effect was not observed with high concentrations of ATPγS, a non-hydrolysable ATP analogue, which showed a dose-dependent inhibitory effect on breast cancer cells. Extracellular ATP is known to be unstable and can be hydrolyzed to other metabolic products, such as ADP, AMP and adenosine. Indeed, prevention of ATP degradation with an ecto-ATPase inhibitor reversed the adverse effect of ATP at higher concentrations. These observations led us to question whether ATP and its degradation product(s) had opposing effect(s) on breast cancer cells. We found that in contrast to the effect by ATP, adenosine increased breast cancer cell migration, and this stimulatory effect was attenuated with an adenosine receptor antagonist. These in vitro cell-based data were validated in vivo using breast cancer mammary fat pad xenograft and intratibial bone metastasis mouse models. This paper, for the first time, uncovers the differential role and the underlying mechanism of ATP and its degradation product, adenosine, in breast cancer growth and metastasis. Therefore, ATP acts as a double-edged sword in cancer growth and metastasis.

The extracellular concentration of adenosine remains constant in most tissues but can rapidly increase 100-fold in response to hypoxia or inflammation, such as in a solid tumor microenvironment, in which its concentrations are increased compared to normal tissue [6]. Adenosine promotes cancer cell migration and chemotaxis in breast cancer and melanoma cells. Furthermore, metastasized cancer cells create a hypoxic microenvironment and express ecto-ATPases. The main pathway leading to high adenosine levels is the hydrolysis of ATP by a family of enzymes known as ectonucleotidases, such as CD39, which hydrolyzes ATP and ADP to AMP and CD73 which hydrolyzes AMP further to adenosine [7]. CD73 has been associated with a pro-metastatic phenotype in breast cancer and knocking down CD73 leads to suppression of breast cancer cell growth, migration and invasion both in vivo and in vitro [8]. Therefore, we must practice caution since the function of ATP on tumorigenesis could largely depend on the activity of ecto-ATPases in the tissue.

ATP and adenosine bind to specific purinergic receptors, which are divided into P1 receptors, with adenosine as the main ligand, and P2 receptors, with ATP and ADP as the main ligands. P2 receptors have seven P2X and eight P2Y subtypes and P1 has A1, A2a, A2b, and A_3_ subtypes. The presence and expression level of the specific P2 or P1 receptor subtypes in cancer cells are key factors determining cellular response to adenosine nucleotides. As shown in our paper, the inhibitory effect on cancer cells by ATP and the stimulatory effect by adenosine were primarily mediated by the activation of P2X_7_ and A_2A_ receptors, respectively and such effects were not seen in breast cancer cell types without these receptors [5]. The cancer cell-specific expression of particular P1 receptor subtypes has been reported in other studies; for example, A2b receptors are absent on ER-positive MCF-7 cells, whereas MDA-MB-231 cells express very high levels of A2b.

**Figure 1:**
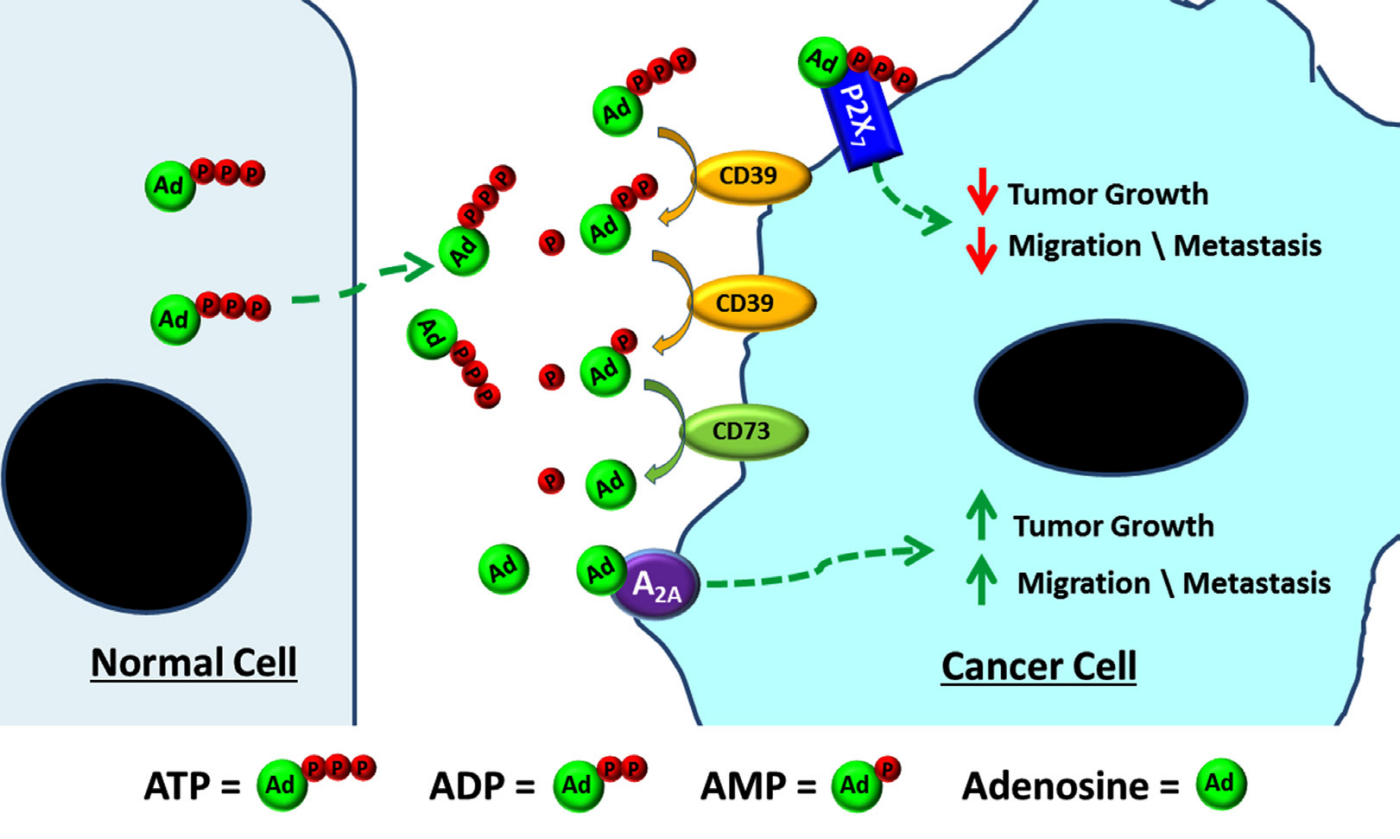
Differential roles of adenosine nucleotides in cancer growth and metastasis ATP released by normal cells (osteocytes] binds to the P2X_7_ receptor and inhibits breast cancer growth, migration and bone metastasis. ATP is unstable and hydrolyzed by ecto-ATPase's, i.e., CD39 and CD73 produced by breast cancer cells. Extracellular ATP is hydrolyzed to ADP and AMP by CD39, and AMP to adenosine by CD73. Adenosine binding to the A2A receptor, which instead promotes cancer cell growth, migration and metastasis.

Based on the evidences provided by our paper and other studies as illustrated in Figure 1, we conclude that it is unsafe to use ATP directly to treat cancer. However, non-hydrolysable ATP analogs could be a viable alternative. Moreover, inhibition of ecto-ATPases could be an effective therapeutic strategy. In fact, anti-CD73 antibody therapy significantly delays tumor growth and lung cancer metastasis in animal models [7]. Furthermore, more targeted therapies to various types of cancers can be achieved through the understanding of unique expression of purinergic receptors and the development of specific agonists and antagonists. This line of research opens up a new opportunity for cancer research and therapeutics.
